# The Impact of Women's Attitudes Toward Intimate Partner Violence and Gender Equity on Intimate Partner Violence Experience: Findings From a Longitudinal Study in Mwanza, Tanzania

**DOI:** 10.1177/10778012251369029

**Published:** 2025-08-21

**Authors:** Joanna Krajewska, Sarah R. Meyer, Neema Mosha, Gerry Mshana, Heidi Stöckl

**Affiliations:** 1 Institute for Medical Information Processing, 9183 Biometry and Epidemiology, Chair of Public Health and Health Services Research, Medical Faculty, Ludwig Maximilian University, Munich, Germany; 2 Pettenkofer School of Public Health, Munich, Germany; 3 Mwanza Intervention Trials Unit, Mwanza, Tanzania; 4 119151 National Institute for Medical Research, Mwanza, Tanzania; 5 Department of Global Health and Development, Faculty of Public Health and Policy, London School of Hygiene and Tropical Medicine, London, UK

**Keywords:** intimate partner violence against women, gender equity, acceptability of intimate partner violence, longitudinal, Tanzania

## Abstract

Inequitable gender norms can manifest in attitudes about gender and intimate partner violence (IPV) acceptability and be risk factors for women's experience of male-perpetrated IPV. This longitudinal study explored the effect of Tanzanian women's attitudes toward gender equity and toward IPV on physical and/or sexual IPV experience with mixed-effects and cross-lagged regressions. Unadjusted mixed-effects regression shows that an increase in gender equity or IPV unacceptance is significantly associated with a decrease of IPV experience. Cross-lagged regression did not find a significant association. Other contextual factors, rather than women's attitudes toward gender equity and IPV, may be key determinants of IPV experiences.

## Introduction

Intimate partner violence (IPV) against women is a significant human rights problem and the most common form of violence against women. It has profound consequences on women's health, safety, and well-being. IPV refers to any physical, sexual, and emotional act of abuse and controlling behaviors by an intimate partner (Sardinha et al., 2022). Recent global estimates indicate that 27% of women over 15 years have been subjected to physical and/or sexual violence by an intimate partner at least once in their lifetime (Sardinha et al., 2022). In Sub-Saharan Africa (SSA), 33% of women have been subjected to physical and/or sexual IPV in their lifetime, making it the WHO region with the second-highest prevalence rate (World Health Organization, 2021).

Traditional gender norms and the acceptability of IPV are well-studied risks for male perpetration and women's experiences of IPV (Krishnan et al., 2012; Sunmola et al., 2020). Promoting gender equity is one of the most important strategies of violence prevention, and shifting attitudes toward the unacceptability of IPV is a central focus of prevention programs (Copp et al., 2019). Many IPV prevention programs focus on changing social norms that sustain violent behavior to create a social environment that promotes gender equity and condemns IPV, e.g., the SASA! Program in Uganda (Abramsky et al., 2014).

The differentiation between social norms and attitudes is important in research and for practitioners who plan to integrate a social norms perspective. Social norms are the perceived informal rules that define acceptable and appropriate actions within a given group or community and that guide human behavior, whereas attitudes refer to an internally motivated judgment about a behavior or practice (Cislaghi & Heise, 2018; United Nations Children's Fund, 2021). Social norms and individual attitudes can coincide or differ (Cislaghi & Heise, 2018). Supportive or accepting attitudes toward IPV may be a risk factor for IPV perpetration and experience. As these are still widespread, they can lead to perceiving IPV as normative, increasing the risk of men's perpetration (Martín-Fernández et al., 2018) and women's justification of IPV, which can increase the risk of IPV occurrence (Ferrer-Perez et al., 2020). Hence, holding positive attitudes toward IPV can put women at risk of experiencing it (Abramsky et al., 2011; Capaldi et al., 2012; Krishnan et al., 2012; Sunmola et al., 2020). However, not conforming to social norms, i.e., having differing attitudes to the social norm, can also be a risk factor for IPV experience. If a social group in a patriarchal society expects women to be submissive toward male authority, violence may be used to punish or “discipline” them if they violate gendered expectations (The Equality Institute, 2017). Some men might use IPV to punish their partner for transgressing gender norms and threatening their masculine role (Weber et al., 2019). Particularly, in the context of marriage, the use of physical violence as a means of behavior correction exists in patriarchal societies. Some husbands may use violence to correct or change their wife's behavior if she does not fulfill culturally expected feminine obligations and duties in marriage or if she is disrespectful or non-submissive toward her husband (Dery, 2019). This corrective use of violence can be justified and seen as “appropriate” by both, perpetrators and victims (Adjei, 2018; Dery, 2019) as it “serves moral and worthy social purposes” (Adjei, 2018, p. 1879) as long as the violence is used in moderation (Adjei, 2018), is not underserved, disproportionately or in anger or drunkenness (Jakobsen, 2014).

This belief, that physical punishment by husbands is justified, could partially explain why women in SSA, when asked about their acceptance of “wife-beating,” show higher acceptance rates than men (Paintsil et al., 2023; Sardinha & Nájera Catalán, 2018). However, the ambiguity and lack of contextual details in direct survey questions, such as the DHS questions on physical IPV, can lead to underreporting of women's actual attitudes and acceptance of physical IPV, especially due to the lack of detail on intentional versus unintentional violation of gendered behavior (Schuler et al., 2011; Tsai et al., 2017). Equally, they can fail to capture the complexity of participants’ actual attitudes, causing them to interpret and answer according to perceived gender norms and socially desirable responses rather than their own personal attitudes (Yount et al., 2013). Also, there is counterintuitive evidence from SSA suggesting that acceptance of IPV among women and girls who have experienced IPV has protective effects, as it was associated with improved mental health and lower odds of suicide ideation (Ibala et al., 2021). Attitudinal acceptance of IPV among those who have experienced IPV may serve as a coping mechanism to reduce stress caused by the cognitive dissonance survivors may experience when their personal beliefs, e.g., disagreement with violence, collide with their reality, e.g., experience of violence (Gilbert & Gordon, 2017; Ibala et al., 2021; Nicholson & Lutz, 2017).

### Conceptual Framework

According to Heise's (2011) ecological framework, IPV is not caused by a single factor, but rather by several factors that interact at and across different levels: an individual's characteristics, a couple's dynamics and couple's conflict solving, community norms, and cultural, economic, and political factors. In the model, gender norms and attitudes toward IPV impact the occurrence of IPV on different levels: gender norms are on the macrosocial and community level, and attitudes toward IPV are on the woman's and her male partner's individual level, as well as on the relationship level. Thereby, the ecological framework relies on a number of theories. In the following, the theories crucial to understanding how social norms and attitudes can promote or discourage violent behavior and define what type of violence is, are discussed in this respect.

Feminist theorists consider gender inequality and sexism as the main causes of IPV and focus on its sociocultural context—patriarchy (Bell & Naugle, 2008). Patriarchy is the systemic dominance of men over women and describes the reproduction of male power at all levels of society, which is manifested through laws, policies, and other forms of regulations (Becker et al., 2022)*.* Violence in patriarchal societies is caused by the belief that men are dominant over women (Magashi & Msenda, 2020). Men's use of violence against women is a means of exerting control and dominance (Dobash & Dobash, 1977). This control is reproduced and maintained in patriarchal societies by social norms and practices that are inherited by generations (Magashi & Msenda, 2020) and are socially accepted, even if men's and women's individual attitudes differ from it.

According to the social learning theory (Akers, 1977; Bandura, 1978), behaviors and values about interpersonal dynamics are learned by observing and reproducing influential role models, such as parents. Direct or indirect exposure to violence from parents during childhood might influence children's attitudes and values on the acceptability of IPV (Bandura, 1978). Copp et al. (2019) show that accepting IPV attitudes are associated with exposure to violence in the family during childhood. Children who have observed violence that was committed by their fathers toward their mothers may be more likely to believe that violence is an appropriate response to a stressful situation. Therefore, experiencing and witnessing violence in childhood increases the likelihood of IPV perpetration (Fine et al., 2021; Jewkes et al., 2021; Mootz et al., 2023; Mulawa et al., 2018), victimization (Li et al., 2019; Shields et al., 2020; Vargas-Fernández et al., 2022), or both (Jung et al., 2019).

Important covariates for both social norms, attitudes toward IPV and IPV experience itself, are drawn from the resource theory (Goode, 1971), which states that IPV occurs due to a lack or an imbalance of resources between partners. Power and control in a relationship are gained through social, personal, and economic key resources. In a lack or an imbalance of resources within a relationship, the partner with less resources might use violence to maintain a dominant position (Basile et al., 2013).

### Tanzania

The United Republic of Tanzania has a lifetime prevalence of physical and/or sexual IPV of 38% and a past 12 months prevalence of 24%, thus falling in the country groups of second highest prevalence worldwide (World Health Organization, 2021). The Tanzanian society is largely patriarchal (Dillip et al., 2018), and prevailing patriarchal gender norms accept women's subordination and justify male violence toward women (Laisser et al., 2011). Strong patriarchal gender norms, specifically male dominance and control, contribute to the normalization of IPV in Tanzania. Powerful social norms hinder women who reject IPV as a “normal” practice from reporting of their IPV experience (McCleary-Sills et al., 2016). Existing studies found high IPV-accepting attitudes in Tanzania among men and women (Krishnan et al., 2012; Meinhart et al., 2020; Reese et al., 2021). According to the Demographic and Health Survey data, 48% of women and 32% of men believed that a husband is justified in beating his wife in at least one of five hypothetical scenarios (Ministry of Health [Tanzania Mainland] et al., 2023). Nevertheless, Laisser et al. (2011) found an increasing questioning of the existing traditional norms regarding male violence against women in Tanzania.

This study addresses a major gap in knowledge by examining the relationship between IPV, attitudes toward gender equity, and attitudes toward IPV over time, as most research has been done cross-sectionally (Abramsky et al., 2020, 2011; Krishnan et al., 2012; Messersmith et al., 2021). A longitudinal perspective can reveal how factors interact and evolve over time, taking changes in IPV prevalence and in attitudes toward gender equity and IPV into account to get closer to identifying causal relationships. This study aims to examine if individual gender equity attitudes and individual attitudes toward IPV are associated with reports of past 12 months physical and/or sexual IPV over time, using cohort data from adult women in Mwanza, Tanzania. First, we describe the prevalence of IPV among participants, their attitudes toward gender equity, and their attitudes toward IPV. We then examine the association between participants’ individual attitudes toward IPV and gender equity on their reports of recent physical and/or sexual IPV experience. We hypothesize that gender equitable attitudes and IPV unaccepting attitudes are significantly negatively associated with past 12-month physical and/or sexual IPV over time, meaning women who are less accepting of violence are also less likely to experience it.

## Methods

### Study Design

The MAISHA longitudinal study followed up the control groups of women participating in the two MAISHA cluster randomized controlled trials (CRTs). The MAISHA trials recruited women through existing micro-finance groups (CRT01) and neighborhood groups (CRT02) to evaluate the impact of an empowerment-based intervention to prevent IPV among women in Mwanza city, Tanzania. Full details of the two CRTs are published elsewhere (Harvey et al., 2021; Kapiga et al., 2019). In both trials, women were interviewed prior to randomization at baseline in 2017 (Wave 1). At the end of trials, women in the control arms were asked to participate in the follow-up study with two additional yearly interviews. Thus, women were interviewed at baseline of the RCTs (Wave 1, *n* = 1,122), at 29 months (trial's endline, Wave 2, *n* = 1,024), 41 months (Wave 3, *n* = 1,008), and 53 months (Wave 4, *n* = 985), using the same questionnaire. The participants were recruited into the study if they were aged 18 and above, residents in Mwanza for at least two years, fluent in Swahili, and consented to participate in the study. This analysis was restricted to women who participated in the first wave of the study and who reported having been in a relationship in the past 12 months due to the outcome variable IPV exposure in the past 12 months. A total of 1,004 control arm women who were in a relationship in the past 12 months participated in Wave 1 (trial baseline), 879 women in Wave 2 (trial follow-up), 854 in Wave 3, and 822 in Wave 4. The retention rate was <82% in all the waves. The main reasons for loss to follow-up were death, migration, or withdrawal from the study.

### Participant and Public Involvement

The MAISHA trials, on which this longitudinal study is based, discussed the purpose of the study with local community leaders and representatives. Throughout the initial trials, the research team worked in collaboration with local community leaders to identify participants and invite them to community information meetings about the CRTs. Community advisory committees, consisting of local and religious leaders, trial participants, and other relevant stakeholders met regularly to facilitate effective communication between the research team and the study communities and to ensure that any concerns raised by community members were addressed promptly.

### Data Collection

Female interviewers trained in interviewing techniques, gender issues, violence and ethical issues related to research on IPV conducted face-to-face interviews in a private setting. A structured questionnaire comprising eight sections (household, women's and partner's characteristics, income, health, attitudes and social norms, relationships and child discipline, partner's childhood and women's community) was used. The questionnaire was developed in English, translated into Swahili, and independently translated back into English. Responses were directly recorded onto tablet computers, and data were uploaded daily to a secure database and reviewed by the data manager. The study was conducted in accordance with WHO recommendations on researching violence against women (World Health Organization, 2001).

The longitudinal study received ethical approval from the Tanzanian National Health Research Ethics Committee (Ref: NIMR/HQ/R.8a/Vol.IX/2475), the Ludwig Maximilian University ethics committee (21-0507), and the London School of Hygiene and Tropical Medicine (Ref: 11918-4).

### Variables

#### Dependent Variable: Reported Past 12 Months Physical and/or Sexual IPV

Reported past 12 months physical and/or sexual IPV experience was measured at each wave using the WHO domestic violence instrument, which has been validated in Tanzania (World Health Organization, 2005). Women who answered “Yes” to any of the act-based questions were defined as having experienced past 12 months physical and/or sexual IPV in this round of data collection. IPV was measured as a continuous variable, using women's frequency answers (*never*—0, *once*—1, *few*—2, *many*—3) to the nine act-based items. A total score between 0 and 27 was calculated for each woman at each round of data collection (see Table 1).

**Table 1.** Questions for Creating the Variables.

Variable	Questions from the questionnaire
Experienced physical and/or sexual violence in the last 12 months	In the past 12 months, has your current partner or any other partner: Slapped you or thrown something at you that could hurt you? Pushed you or shoved you or pulled your hair? Hit you with his fist or with something else that could hurt you? Kicked you, dragged you, or beaten you up? Choked or burnt you on purpose? Threatened to use or actually used a gun, knife, or other weapon against you? Forced you to have sexual intercourse by threatening you, holding you down, or hurting you in some way? Had sexual intercourse when you did not want to because you were afraid that your partner would hurt you or someone you cared about if you refused? Had sexual intercourse when you did not want to because you were afraid that your partner would leave you or take another girlfriend if you refused?
Attitudes toward gender equity	It's a wife's obligation to have sex with her husband even if she doesn’t want to. It must be the man who is the primary provider for the family. A woman should obey her husband's wishes even if she disagrees. Even healthy relationships can include hitting each other as long as the partners love each other. The leadership of a community should be largely in the hands of men. It is natural and right that men have more power than woman in the family.
Attitudes toward IPV	A man has a good reason to hit his wife if she does not complete her household work to his satisfaction. A man has a good reason to hit his wife if she disobeys him. A man has a good reason to hit his wife if she refuses to have sexual intercourse with him. A man does not have any reason to hit his wife in any way (recoded). A man has a good reason to hit his wife if she protests because he has other girlfriends. A man has a good reason to hit wife if he suspects she is unfaithful in marriage. A man has a good reason to hit wife if he finds out that she has been unfaithful in marriage.
Experienced physical and/or sexual violence in childhood	During the first 15 years of your life, did a parent, or other adult in the household ever Call you bad words, insult you or put you down? Threaten you with physical harm? Spank, slap, kick, punch or beat you up? Hit you so hard that you had marks or were injured? Did an adult or person at least 5 years older than you touch or fondle you in a sexual way? Make you touch their body in a sexual way? Attempt oral, anal, or vaginal intercourse with you? Actually have oral, anal, or vaginal intercourse with you?
Witnessed parental IPV in childhood	When you were growing up, during the first 15 years of your life did you see or hear a parent or household member in your home being slapped, kicked, punched or beaten with a fist or object?
Social economic status quantile	The socio-economic status indicator was derived as a latent variable from 19 indicators collected in the questionnaire including education, earnings and household ownership of car, fridge, television and motorcycle. A higher quintile indicates higher socio-economic status.
More contribution than husband	Would you say that the money that you bring into the household is more than what your husband/partner contributes, less than what he contributes, or about the same as he contributes?

#### Independent Variables

Exposure 1: Attitudes toward gender equity. Women's attitudes toward gender equity were measured with six questions that were developed jointly with the local team through discussions to fit the local context and closely aligned to questions from the gender equitable men (GEM) scale (Pulerwitz & Barker, 2008), and a four-point Likert scale at each wave (see Table 1). The scale's reliability was medium at each wave (Cronbach's alpha at Waves 1 and 2: 0.68, Wave 3: 0.63, and Wave 4: 0.66). An additive scale variable was created. For each of the six questions, women were assigned the following points: 0—*I strongly agree*, 1—*I agree*, 2—*I disagree*, and 3—*I strongly disagree*. The survey scale was then standardized to a range of 0–3.

Exposure 2: Attitudes toward unacceptance of IPV. Women's unaccepting attitudes toward IPV were measured with seven questions, closely aligned to questions on women's attitudes toward violence from the WHO multi-country study on women's health and domestic violence against women (World Health Organization, 2005) and a four-point Likert scale at each wave (see Table 1). The scale's reliability was high at each wave (Cronbach's alpha at Waves 1 and 2: 0.80, Wave 3: 0.81; Wave 4: 0.78). An additive scale variable was created. For each of the seven questions, women were assigned the following points: 0—*I strongly agree*, 1—*I agree*, 2—*I disagree*, and 3—*I strongly disagree*. The survey scale was then standardized to a range of 0–3.

#### Covariates

The inclusion of covariates was guided by the literature review, especially the social learning theory and the resource theory (Basile et al., 2013; Copp et al., 2019; Heise, 2011; Kinyondo et al., 2021; McCleary-Sills et al., 2016). We included the following covariates: age (continuous variable), frequency of having experienced physical and/or sexual violence in childhood by a household member (continuous variable), having witnessed parental IPV in childhood (binary variable), women's social economic status (SES) (categorical variable with five quantiles) and women's income contribution to the household compared to their partners (categorical variable—contributes more, about the same, less than partner) (see Table 1).

Having experienced physical and/or sexual violence in childhood by a household member and having witnessed parental IPV in childhood were only collected at Wave 1. Assuming that those variables are time invariant, we imputed the answer from Wave 1 into the following waves. Age was collected at Wave 1 and Wave 4. To report women's age correctly, we imputed the answer from Wave 1 into the other waves, adding two years to the reported age for Wave 2 and three years for Wave 3. SES quantiles were collected at Wave 1 and Wave 4. To allow for changes in the variables, we decided to impute answers from Wave 1 into Wave 2 and answers from Wave 4 into Wave 3.

### Statistical Analysis

#### Aim 1: Description of Participants’ Prevalence of IPV, Their Attitudes Toward Gender Equity and Their Attitudes Toward IPV

First, we described participants’ prevalence of IPV, their attitudes toward gender equity, and their attitudes toward IPV. A *t*-test for the continuous variable “age” and the scale variables “attitudes toward gender equity” and “attitudes toward IPV unacceptance” and a Pearson Chi-square test for the remaining categorical variables were conducted to assess the mean and proportion difference among women who reported physical and/or sexual IPV versus women who did not report IPV for each covariate at baseline (see Table 2). To visualize the prevalence for IPV and the distribution of attitudes toward gender equity and attitudes toward IPV unacceptance, bar charts and boxplots were created for each wave. Statistically significant changes between the four waves were checked by conducting the Wilcoxon signed rank test for continuous scale variables “physical and/or sexual IPV frequency,” “attitudes toward gender equity,” and “attitudes toward IPV unacceptance.”

**Table 2.** Participant Characteristics at Baseline Associated With Past 12-Month Physical and/or Sexual IPV.

	Total	No physical or sexual IPV experience in the past 12 months	Physical and/or sexual IPV experience in the past 12 months	*p*-value
	*N* = 1,004	*N* = 667	*N* = 337	
Women's age, *M* (*SD*)	35.2 (8.9)	36.1 (9.3)	33.5 (7.9)	<.001
Experienced violence in childhood by household member, *Mdn* (IQR)	3.0 (0.0–6.0)	2.0 (0.0–5.0)	4.0 (1.0–7.0)	<.001
Witnessed parental IPV in childhood				.029
Yes	66.3%	64.0%	70.9%	
No	33.7%	36.0%	29.1%	
Social economic status levels				.003
1st quintile	23.6%	20.7%	29.4%	
2nd quintile	21.3%	19.9%	24.0%	
3rd quintile	23.5%	25.6%	19.3%	
4th quintile	17.4%	18.9%	14.5%	
5th quintile	14.1%	14.8%	12.8%	
More contribution than husband				.006
Less than partner	64.2%	67.5%	57.9%	
Same as partner	6.5%	6.4%	6.5%	
More than partner	29.3%	26.1%	35.6%	
Gender equity score, *Mdn* (IQR)	1.8 (1.3–2.3)	1.8 (1.3–2.3)	1.8 (1.3–2.3)	.093
IPV unacceptance score, *Mdn* (IQR)	2.3 (1.7–2.9)	2.3 (1.7–2.9)	2.1 (1.7–2.7)	.014

*Note*. IPV = intimate partner violence; IQR = interquartile range.

#### Aim 2: Association Between Participants’ Individual Attitudes Toward IPV and Gender Equity on Their Reports of Recent Physical and/or Sexual IPV Experience

We then examined the association between participants’ individual attitudes toward IPV and gender equity on their reports of recent physical and/or sexual IPV experience, hypothesizing that gender equitable and IPV-unaccepting attitudes would be significantly negatively associated with recent physical and/or sexual IPV over time. A mixed-effects linear regression model was fitted with the continuous outcome physical and/or sexual IPV experience, and the pre-defined covariates to disentangle the within-participant effects from the between-participant effects in the panel data. Participant's ID was used as a random effects parameter. The mixed-effects linear regression assumptions were met, and no high correlation between variables was detected (for most variables Spearman correlation coefficient < 0.3). Moderate correlation was detected between attitudes toward gender equity and attitudes toward IPV (Spearman correlation coefficient < 0.44 at each wave). To control for confounders, unadjusted and adjusted mixed-effects models with all waves were compared. Six different models were built. Model 1 included attitudes toward gender equity only (unadjusted), Model 2 included attitudes toward gender equity and the confounders (adjusted). Model 3 included attitudes toward IPV only (unadjusted), Model 4 included attitudes toward IPV and the confounders (adjusted). Model 5 included both attitude variables (partly adjusted) and Model 6 included all independent variables (fully adjusted). To control for non-responses being at random, a sensitivity analysis for the final mixed-effects regression with both attitude variables as main exposure was conducted, using only women who participated in all four waves. The outcome direction did not change; therefore, missing at random is assumed. A sensitivity analysis for the scale variables “attitudes toward gender equity” and “attitudes toward IPV unacceptance” was conducted through conceptualizing the variables as categorical variables. The outcome direction was similar. To show more precise information, the scale variables were used.

Next, the longitudinal data was examined with a cross-lagged panel regressions to investigate the effect of previous waves’ (*t* − 1) physical and/or sexual IPV experience, attitudes toward gender equity (Model 7), attitudes toward IPV (Model 8), and both (Model 9) on the subsequent wave's physical and/or sexual IPV experience (*t*). To simultaneously control for unobserved confounders and to include lagged, endogenous regressors, we have used maximum likelihood structural equation modeling (Williams et al., 2018) and the Satorra–Bentler adjustment to adjust for non-normally distributed variables.

The results of all linear regression analyses are presented as coefficients (*β*) with standard errors (*SE*). Statistical significance was defined as a two-tailed *p*-value < .05 in all analyses. All analyses were conducted in STATA Version 17 SE (StataCorp., TX, USA).

## Results

### Aim 1: Description of Participants’ Prevalence of IPV, Their Attitudes Toward Gender Equity, and Their Attitudes Toward IPV

Participant characteristics at baseline (*n* = 1,004), including attitudes toward gender equity, attitudes toward IPV, socio-demographic and economic background, and violence experiences in childhood, are shown in Table 2. At baseline, women were 35 years old on average. Their median level of childhood violence experience by a household member was 3.0 (IQR 6.0), and 66.3% witnessed parental IPV. More than half (64.2%) contributed less income than their partner to the household. The median gender equity score was 1.8 (IQR 0.99), and the median IPV unacceptance score was 2.3 (IQR 1.1).

At baseline, 33.7% women reported one or more acts of physical and/or sexual IPV in the past 12 months. Those women were younger on average (33.5 years) (*p* < .001) than women who did not report IPV. Women who reported IPV had a higher median on the violence experience during childhood by a household member frequency scale (4.0, IQR 6.0) than women who did not experience IPV (*p* < .001) and had higher proportions of having witnessed parental IPV in childhood (70.9%) (*p* = .029). Half of the women who reported IPV (57.9%) contributed less to the household income than their partner, compared to 67.5% of women who did not report IPV. Also, women who reported IPV had higher proportions of contributing more than their partner to the household income (35.6%) than women who did not report IPV (26.1%) (*p* = .006). Women who reported IPV showed similar attitudes toward gender equity (*Mdn* 1.8, IQR 0.99) as women who did not report IPV (*Mdn* 1.8, IQR 0.99). Also, women who reported IPV had a similar but significantly lower median score on the “attitudes toward IPV unacceptance” scale (*Mdn* 2.1, IQR 1.1) compared to women who did not report IPV (*Mdn* 2.3, IQR: 1.1).

**Figure 1.**
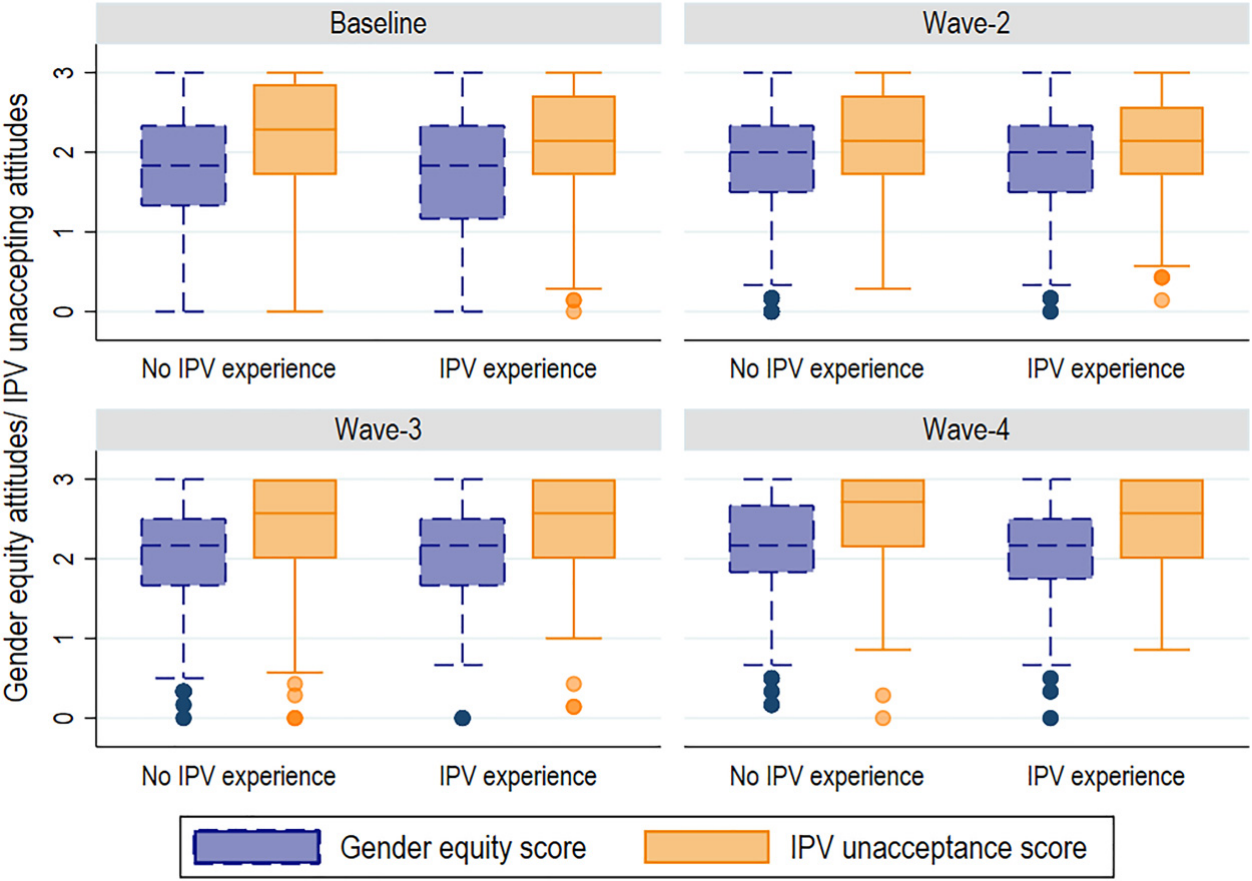
Distribution of Women's Scores of Attitudes Toward Gender Equity and Toward IPV Unacceptance According to IPV Experience, by Waves.

*Note*. IPV = intimate partner violence.

Descriptive analysis shows that participants’ reports of physical and/or sexual IPV experience in the past 12 months decreased over time, with a significant change in medians between Waves 1 and 4, and participants’ median score on the gender equitable attitudes and IPV unaccepting attitudes scale shifted toward a higher score (see Figure 1 and Supplemental material 1). Over time, increasingly more women, those who experienced IPV and those who did not, showed higher scores on the gender equitable attitudes scale and on the IPV unacceptance scale (see Figure 1). Women who experienced IPV and women who did not experience IPV in the past 12 months showed similar median scores on the gender equity scale in all waves and similar median scores on the IPV unacceptance scale in the first three waves. Only in the first and last wave, women who experienced IPV showed a significantly lower median score on the IPV unacceptance scale (2.14 at visit 1; 2.57 at visit 4) than women who did not experience IPV (2.28 at Visit 1; 2.71 at Visit 4) (see Figure 1).

### Aim 2: Association Between Participants’ Individual Attitudes Toward IPV and Gender Equity on Their Reports of Recent Physical and/or Sexual IPV Experience

In the bivariate model, “having witnessed parental IPV during childhood” was not significantly associated with the outcome. Once “experienced physical and/or sexual violence in childhood” or “women's age” was added to the model, the association became significant. However, “having witnessed parental IPV during childhood” was kept in the final model to reflect the conceptual framework of this analysis.

Unadjusted and adjusted mixed-effects linear regressions are shown in Table 3. In the bivariate model, an increase of one point on the gender equity scale is associated with an expected decrease of −0.24 in IPV frequency (coefficient: −0.24, *SE*: 0.1). By adding IPV unaccepting attitudes and all other covariates, the association becomes insignificant. The same effect can be seen for IPV unaccepting attitudes. In the bivariate model an increase of one point on the IPV attitudes scale, indicating an increase in attitudes rejecting IPV, is associated to a decrease in IPV frequency by 0.36 (coefficient: −0.36, *SE*: 0.1). By adding gender equity attitudes into the model, the expected change decreases to 0.31 (coefficient: −0.31, *SE*: 0.12). However, by adding all covariates, the association becomes insignificant, disproving our initial hypothesis that gender equitable attitudes and IPV-unaccepting attitudes are significantly negatively associated with recent physical and/or sexual IPV.

**Table 3.** Unadjusted and Adjusted Mixed-Effects Logistic Regressions With Reports of Physical and/or Sexual IPV as Dependent Variable.

	Model 1 Unadjusted	Model 2 Adjusted	Model 3 Unadjusted	Model 4 Adjusted	Model 5 Unadjusted	Model 6 Fully adjusted
Variables^a^	IPV	IPV	IPV	IPV	IPV	IPV
Gender equity score, *β (Standard error)*	−0.24* (0.10)	−0.02 (0.11)			−0.10 (0.12)	0.03 (0.12)
IPV unacceptance score, *β (Standard error)*			−0.36** (0.10)	−0.11 (0.11)	−0.31** (0.12)	−0.12 (0.12)

*Note*. IPV = intimate partner violence; SES = social economic status. 3,559 individual observations and 1,054 groups for each model.

**p < .01, *p < .05

(a) Variables in the regression model:
in all models: participant's ID as random-effect intercept. unadjusted model: only the variable of interest (attitudes toward gender equity, attitudes toward IPV or both). adjusted model: the variable of interest and controlling for: age, frequency of experienced violence in childhood, witnessed parental IPV in childhood, SES quantile and income contribution to household compared to partner

In the final Model 6 (see Supplemental material 2), several covariables were statistically significantly associated to the outcome: women's age (coefficient: −0.04, *SE*: 0.01), frequency of experienced violence in childhood (coefficient: 0.17, *SE*: 0.03), having witnessed parental IPV (coefficient: −0.72, *SE*: 0.20), SES quintile 3 (coefficient: −0.58, *SE*: 0.22), 4 (coefficient: −0.87, *SE* 0.23), 5 (coefficient: −0.93, *SE*: 0.25), more contribution to the household income than partner (coefficient: 0.61, *SE*: 0.15), the time-variables Wave 2 (coefficient: −0.40, *SE*: 0.15), 3 (coefficient: −0.73, *SE*: 0.15), and 4 (coefficient: −0.76, *SE*: 0.16). The estimated variance of the constant per person—the variance between individuals—was 5.86 (*SE* 0.40).

The intra-class correlation (ICC) for the fully adjusted model (Model 6) was 0.38 (*SE*: 0.02). Hence, 38% of the total variance in IPV in the model was explained by individual differences between women, while 62% of the variation in IPV was explained by differences in observations by wave or over time within the same woman. The Akaike information criterion (AIC) was lower for the fully adjusted model (AIC 19352.58) than for the intercept-only model (AIC 19501.55). The mixed-effects linear regression model was a significantly better fit than a simple regression model.

Unadjusted and adjusted cross-lagged panel regression using maximum likelihood structural equation modeling are shown in Table 4. No significant associations between previous wave's IPV experience and subsequent IPV experience, previous wave's attitudes toward gender equity and subsequent IPV experience as well as previous wave's attitudes toward IPV and subsequent IPV experience were proven in the models. Hence, no longitudinal relation between women's attitudes toward gender equity, their attitudes toward IPV, and IPV experience was found in the cross-lagged panel data regression.

**Table 4.** Unadjusted and Adjusted Dynamic Panel Regression With Reports of Physical and/or Sexual IPV as Dependent Variable.

	Model 7 Unadjusted	Model 8 Unadjusted	Model 9 Partly adjusted
Variables			
IPV (physical and/or sexual) frequency *t* − 1^b^, *β (Standard error)*	0.07 (0.06)	0.07 (0.06)	0.07 (0.06)
Gender equity *t* − 1^b^, *β (Standard error)*		−0.12 (0.16)	−0.05 (0.17)
IPV unacceptance score *t* − 1^b^, *β (Standard error)*	−0.26 (0.18)		−0.24 (0.19)
Alpha, *β (Standard error)*	1.00 (0.00)	1.00 (0.00)	1.00 (0.00)

*Note*. IPV = intimate partner violence. 707 observations for each model.

**p < .01, *p < .05.

(b) For Waves 2, 3, and 4.

## Discussion

In this longitudinal study, a significant relationship in two unadjusted models emerged: when women had higher levels of attitudes supporting gender equity, they were less likely to have experienced physical and/or sexual IPV, and when women had attitudes rejecting IPV perpetration they were less likely to have experienced physical and/or sexual IPV across the course of the study. When adjusting for covariates, these associations became insignificant. No significant association between those factors was found in the dynamic panel data regression.

Regarding the prevalence rates of IPV, of attitudes toward gender equity and of attitudes toward IPV, our findings are similar to other studies. Past 12 months physical and/or sexual IPV prevalence in Tanzania was measured by DHS data of 26.4% (Ministry of Health [Tanzania Mainland] et al., 2023). This is comparable to the prevalence rate in the first two waves (23.8% at baseline, 22.2% in Wave 2) of this study, which declined in the following waves. Our findings of attitudes toward IPV are difficult to compare with findings from the DHS reports due to different measurements. The Tanzanian DHS (Ministry of Health [Tanzania Mainland] et al., 2023) reports that 52% of women do not justify wife beating in any of five hypothetical scenarios. Our study population showed a median score of IPV unacceptance on a scale from 0–3 of 2.28 in Wave 1 and 2.14 in Wave 2, which increased in the following waves (Wave 3 and 4: 2.57). Attitudes toward gender equity are often measured using standardized questions from the GEM scale (Pulerwitz & Barker, 2008). The IMAGES study showed that in Tanzania, the average GEM scale score for women on gender equity is 1.69 on a range from 0 to 3 (Levtov et al., 2018). Our findings, with a slightly different set of questions, showed that the median gender equity score on a scale from 0 to 3 was similar to IMAGES findings in the first wave (1.83) and increased in the following (Wave 2: 2.0, Wave 3 and Wave 4: 2.16).

Due to the panel data in our study, we can see that over time, less physical and/or sexual IPV was reported, and women showed more gender equitable and more IPV-unaccepting attitudes. Changes in Tanzanian women's attitudes toward wife beating over time are also reported in DHS data. DHS data from 2022 (Ministry of Health [Tanzania Mainland] et al., 2023) showed a decrease in women's attitudes on justifying wife beating to 48% from prior 58% in 2016 (Ministry of Health, Community Development, Gender, Elderly and Children [Tanzania Mainland] et al., 2016). However, bias in our results is possible. Repeated exposure to the same questions about gender equity and IPV over the course of the study might have impacted participants’ answer. Also, intervention contamination of the study population during the first two waves of this longitudinal study might have influenced participants’ answers as the intervention group of the MAISHA trials received sessions on gender training, gender norms, and attitudes.

Our findings of the negative associations between attitudes toward gender equity and IPV unacceptance on IPV experience in the bivariate models are in line with the general scientific consensus. Theories, e.g., feminist theories or the social learning theory, and empirical cross-sectional studies have shown a positive significant effect between women's gender inequity attitudes and reports of IPV (Corley et al., 2021; Krishnan et al., 2012) and between women's IPV-accepting attitudes and reports of IPV (Abramsky et al., 2020, 2011; Sunmola et al., 2020; World Health Organization & London School of Hygiene and Tropical Medicine, 2010). Nevertheless, adjusting for both attitudes variables, these associations became insignificant. This insignificant association in the fully adjusted model indicates that factors other than women's attitudes toward gender equity and IPV have a stronger effect on women's IPV experience. Higher age, more frequent experience of violence during childhood, witnessing parental IPV, the highest SES quintile, contributing more income to the household than the partner, and the time factor showed a significant association with reports of IPV in the mixed-effects regression model and are known factors that affect women's IPV experience. This suggests that women's attitudes toward gender equity and IPV may not be key determinant of IPV experience on its own, but rather contextual factors, such as their partner's or society's attitudes and norms. Men's gender inequitable and IPV-accepting attitudes are known to be significantly associated with IPV perpetration (Fleming et al., 2015; McNaughton Reyes et al., 2022; Messersmith et al., 2021).

Only targeting women's individual attitudes toward gender equity and IPV acceptance in prevention interventions may therefore not be sufficient to reduce levels of IPV. Within a community and in real-life situations, individuals do not always behave according to their individual attitudes as they act within community norms and expectations and align with them for different reasons. This is in line with an observable change in violence prevention practice that has evolved from programs targeting individual women to approaches that focus on shifting gender norms in communities and including men and boys to transform norms that foster gender inequality and violence (Fine et al., 2021; Jewkes et al., 2015). Generally, IPV interventions including men were more effective than those targeting only women (Alsina et al., 2023; Jewkes et al., 2021; Leight et al., 2023). Leight et al. (2023) found in their systematic review that gender transformative interventions at the community-level or group-based interventions were effective and reduced the odds of women's IPV experience in the past year. Semahegn et al. (2019) also recommend community gender-norm transformation through an intensive information, education, and communication (IEC) approach, among others, as interventions to prevent IPV. A prominent and successful transformative gender norms intervention is the SASA! Program in Uganda (Abramsky et al., 2014). It showed significant effects on women's IPV acceptance levels, reductions in women's past year experience of physical IPV and in men's perpetration of IPV (Abramsky et al., 2016). IPV interventions should not only address social norms around violence and gender alone, as successful violence prevention programs address multiple drivers of violence (Jewkes et al., 2021).

No interaction between gender equity and IPV unacceptance attitudes was identified, and the correlation was moderate (Pearson's R 0.48–0.5 at each wave). However, we suspect multicollinearity between the two variables as the variable gender equity turns quickly insignificant when adding other variables to the model. In a causal model, gender equity attitudes could be a potential mediator between IPV-unaccepting attitudes and IPV experience. The stronger association with IPV unacceptance and IPV experience might be due to more narrow questions on IPV acceptance than on gender equity. Fine et al. (2021) discuss that a narrower focus on IPV acceptance in prevention interventions, especially among participants who have already experienced IPV, might have a greater impact on reducing IPV than a broader scope on shifting norms and attitudes around gender equity.

This study measured individual-level attitudes. It is possible that participants’ individual attitudes are not reflective of their actual actions and behavior once surrounding social norms are strong. A better understanding of individual attitudes as risk factors of IPV could be achieved through exploring and comparing community-level attitudes and measuring social norms. Factors such as social norms on gender equity, which are reflected in laws, policies, and regulations of formal social institutions, might have a stronger association with IPV. One could examine whether women's attitudes toward gender equity align with social norms and analyze which women show higher or lower odds for reports of IPV. Examples of this can be seen in the work of Shakya et al. (2022) or McGhee et al. (2021). In general, it is highly recommended to distinguish in measurements between social norms and attitudes (Cislaghi & Heise, 2018; Learning Collaborative to Advance Normative Change, 2019).

It is plausible that several limitations may have influenced our results. Given the sampling procedures, the results may not be generalizable beyond women in Mwanza, Tanzania. Although in the mixed-effects regression we controlled for unmeasurable confounders within the same participants through the random effect coefficient, possibly omitted confounders or colliders might have affected the association of the variables. Not differentiating between different types of IPV, sexual, physical, but also emotional and economic, nor the concrete frequency of IPV, may have prevented us from observing differences in the associations between attitudes toward gender equity and IPV and reports of IPV. Also, the missing context or motivation behind the violence experiences and not accounting for women's self-reported perpetration of IPV limit our findings. It is possible that some of the participants were violent toward their partner or reacted violently to experiencing violent behavior. Acceptance or justification of wife beating has been shown to be associated with female IPV perpetration in Tanzania (Reese et al., 2021).

Further research should focus on which factors other than women's attitudes have an influence on the relation between women's IPV experience and social norms regarding IPV. Here, the differentiation in measurements between social norms and individual attitudes should be noted, and both should be assessed. We suspect from our findings that IPV prevention programs that aim to change social norms toward IPV should not solely focus on the individual women's attitudes, but rather also on the interpersonal and community level, include men as well, and address multiple risk factors in order to shift traditional community gender norms and expectations in which individuals interact.

## Conclusion

This study established a univariate relationship between women's attitudes toward gender equity, toward IPV, and IPV experience in the bivariate mixed-effects models that disappeared once other factors were included in the model. Also, no longitudinal relationship was found in the cross-lagged panel regression. This suggests that women's attitudes toward gender equity and IPV may not be the key determinant of IPV experience on its own, but rather contextual factors, such as their partner's or communities’ attitudes and norms. Further research should focus on which factors other than women's attitudes have an influence on the relation between women's IPV experience and social norms regarding IPV. A clearer understanding of the role of attitudes and norms on gender equity and IPV, in relation to other factors, would strengthen the evidence base for developing and testing appropriate IPV interventions in settings such as Tanzania.

## Supplemental Material

sj-docx-1-vaw-10.1177_10778012251369029 - Supplemental material for The Impact of Women's Attitudes Toward Intimate Partner Violence and Gender Equity on Intimate Partner Violence Experience: Findings From a Longitudinal Study in Mwanza, Tanzania Supplemental material, sj-docx-1-vaw-10.1177_10778012251369029 for The Impact of Women's Attitudes Toward Intimate Partner Violence and Gender Equity on Intimate Partner Violence Experience: Findings From a Longitudinal Study in Mwanza, Tanzania by Joanna Krajewska, Sarah R. Meyer, Neema Mosha, Gerry Mshana and Heidi Stöckl in Violence Against Women
