# Supplementary material for: The Impact of Women's Attitudes Toward Intimate Partner Violence and Gender Equity on Intimate Partner Violence Experience: Findings From a Longitudinal Study in Mwanza, Tanzania
Source: Violence Against Women. 2025 Aug 21;32(11):4042–66. doi: 10.1177/10778012251369029 (PMC13332167; doi:10.1177/10778012251369029)
Supplement: sj-docx-1-vaw-10.1177_10778012251369029 - Supplemental material for The Impact of Women's Attitudes Toward Intimate Partner Violence and Gender Equity on Intimate Partner Violence Experience: Findings From a Longitudinal Study in Mwanza, Tanzania [file sj-docx-1-vaw-10.1177_10778012251369029.docx]

**Supplementary material**

Supplementary material 1: Experienced past 12 months physical and/or sexual IPV, by waves


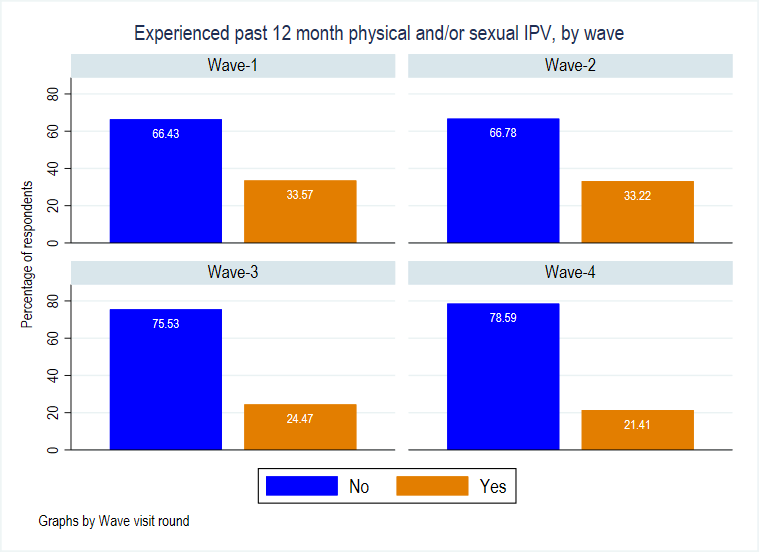


Supplementary material 2: Fully adjusted mixed-effects linear regression (Model 6)

| Variables | Model 6, fixed effects  Coefficient, (SE) |
| --- | --- |
| Gender equity attitudes score | 0.03 |
| *Standard Error* | (0.12) |
| IPV unacceptance score | -0.12 |
| *Standard Error* | (0.12) |
| Women's age | -0.04** |
| *Standard Error* | (0.01) |
| Experienced violence during childhood frequency score | 0.17** |
| *Standard Error* | (0.03) |
| Not witnessed parental IPV in childhood | 1 |
| *Standard Error* | / |
| Witnessed parental IPV in childhood | -0.72** |
| *Standard Error* | (0.20) |
| Social economic status level 1^st^ quintile | 1 |
| *Standard Error* | / |
| Social economic status level 2nd quintile | -0.38 |
| *Standard Error* | (0.22) |
| Social economic status level 3rd quintile | -0.58** |
| *Standard Error* | (0.22) |
| Social economic status level 4th quintile | -0.87** |
| *Standard Error* | (0.23) |
| Social economic status level 5th quintile | -0.93** |
| *Standard Error* | (0.25) |
| Less income contribution to household than partner/husband | 1 |
| *Standard Error* | / |
| Same income contribution to household as partner/husband | 0.01 |
| *Standard Error* | (0.24) |
| More income contribution to household than partner/husband | 0.61** |
| *Standard Error* | (0.15) |
| Wave1-visit | 1 |
| *Standard Error* | / |
| Wave-2 visit | -0.40** |
| *Standard Error* | (0.15) |
| Wave-3 visit | -0.73** |
| *Standard Error* | (0.15) |
| Wave-4 visit | -0.76** |
| *Standard Error* | (0.16) |
| Regression constant | 4.36 |
| *Standard Error* | (0.50) |
| Observations | 3,559 |
| Number of groups | 1,054 |

Variance coefficient of Model 6

| Variance of individual women between waves (estimate) | 5.86 |
| --- | --- |
| *Standard Error* | (0.40) |
| Variance of residuals of individual women between waves (estimate) | 9.66 |
| *Standard Error* | (0.27) |
